# Influence of the Quartz Deformation Structures for the Occurrence of the Alkali–Silica Reaction

**DOI:** 10.3390/ma11091692

**Published:** 2018-09-12

**Authors:** Francieli Tiecher, Renata N. Florindo, Geilma L. Vieira, Márcia E. B. Gomes, Denise C. C. Dal Molin, Richard T. Lermen

**Affiliations:** 1 Polytechnic School of Civil Engineering, Faculdade Meridional IMED, Passo Fundo 99070-220, Brazil; richard.lermen@imed.edu.br; 2 Instituto de Física de São Carlos, Universidade de São Paulo, São Carlos 13566-590, Brazil; rflorindo@gmail.com; 3 Department of Civil Engineering, Universidade Federal do Espírito Santo, Vitória 29075-910, Brazil; geilma.vieira@gmail.com; 4 Department of Geology, Universidade Federal do Rio Grande do Sul, Porto Alegre 90040-060, Brazil; marcia.boscato@ufrgs.br; 5 Department of Civil Engineering, Universidade Federal do Rio Grande do Sul, Porto Alegre 90040-060, Brazil; dmolin@ufrgs.br

**Keywords:** alkali–silica reaction, quartz, silica dissolution

## Abstract

Defects in the crystalline structure of quartz facilitate the connection with the alkali hydroxides, since under a high alkalinity condition (e.g., in concrete), the Si-O bonds of quartz are easily broken. This study set out to investigate the influence of the deformation structures of quartz on its susceptibility to the alkali–silica reaction. A granite, a protomylonite, and a mylonite were selected for this study. Using optical microscopy, the quartz grains contained in these rocks were quantified and their texture characterized. The quartz samples extracted from the rocks were analyzed by magnetic nuclear resonance, to evaluate their potential for dissolving silica as well as changes in their atomic scale before and after the reaction with alkali hydroxides. These analyses were compared with the results of the accelerated mortar bar test. The study showed that the quartz with intense undulatory extinction and deformation bands denotes the most favorable condition to the development of the alkali–silica reaction. However, on an atomic scale, the slightly deformed grains were highly prone to react. Thus, in a high alkalinity condition, over a long period of time, any quartz tends to develop the alkali–silica reaction, regardless of the deformation degree of the grain.

## 1. Introduction

Quartz is the main mineral responsible for triggering the alkali–silica reaction (ASR) in concrete structures [1,2,3]. This mineral has a tetrahedral coordination of silicon (Si) by oxygen (O) stable, but due to tectonic stresses its crystalline structure undergoes displacements and deflections [1]. The displacement of the crystalline structure of quartz leads to changing the distances of its atoms, thus creating “fragile zones” which, due to the increase in internal tensions, tends to subdivide and form new grains [4]. Its crystal lattice becomes slightly bent and this can be observed under a petrographic microscope. By virtue of the undulatory extinction, this characteristic indicates the greater reactivity of quartz in alkali hydroxides of concrete. Wenk et al. [5] and Tiecher et al. [6] observed the formation of displacement walls in quartz grains by using transmission electron microscopy (TEM), thereby verifying that the displacements of the crystalline reticulum occur in small areas, where walls that divide the crystal into subgrains are formed, and that these have little or no displacement.

Therefore, the same grain could have zones that are more and less reactive, and which are directly related to the thermodynamic stability of the tensioned quartz to the alkaline solution as it can attack the mineral in different ways, in accordance with its degree of deformation. Thermodynamically unstable quartz with high free energy in its crystalline networks is found in very deformed granites and in metamorphic rocks [7]. However, metamorphic aggregates are considered “slow” to develop the ASR [8,9,10,11]. The reduction in grain size during metamorphic deformation increases the specific surface area enhancing the reaction. However, the new grains are not always deformed and may take longer to react [12].

This study evaluates the susceptibility of quartz to react with alkaline solutions, as a function of degree of deformation with quartz grains.

## 2. Materials and Methods

### 2.1. Selection of the Samples

The samples used in this study are the same as those of Tiecher et al. [6]. A specific experimental procedure was employed in order to evaluate the influence of deformation intensity of quartz crystals on ASR. A granitic rock that records the progression from magmatic features to solid-state deformation in a ductile shear zone was selected for this study. Representative samples of the granite and of two deformation conditions, named protomylonite and mylonite were collected. The sample selection targeted to provide quartz with different deformation structures. The outcrop is located in Monte Bonito, in the municipality of Pelotas, in the south of the state of Rio Grande do Sul, which is part of the Pelotas Batholith.

The Pelotas Batholith is a plutonic complex with approximately 270 km of length and 70–110 km of width that crops out in the eastern portion of the South-Riogrande Shield and continues into Santa Catarina and Uruguay [13]. It comprises seven granitoid suites which are cut by extensive NE shear zones, generating varied mylonitic rocks, from protomylonites to ultramylonites [14,15,16,17,18,19,20].

In the outcrop, it was possible to identify three degrees of deformation of the rock, which were called M1, M2, and M3, where M1 represents the rock with the least deformed minerals, M2 the rock with intermediate deformation, and M3 the rock with the most deformed minerals.

### 2.2. Preparation of the Samples

In order to evaluate the influence of the quartz deformation on the products formed by ASR, without the interference of other silicates present in the samples, the quartz grains were extracted from the matrix of the rocks.

First, samples were ground to obtain grains smaller than 0.15 mm, because this is the average size of most minerals from the rocks. In this process some grains can be subdivided, but their crystalline structure does not change; in this way it is assumed that the behavior and results obtained will not be affected by the comminution. After that, the minerals were separated magnetically to extract those containing iron (predominantly micas).

The separation between quartz and feldspar (K-feldspar and plagioclase) was done using the density difference in a volumetric flask. The ground rock was immersed, without the micas, in bromoform (CHBr3-density 2.84 g/cm^3^), adding acetone (CH_3_(CO)CH3-99.5%, density 0.788 g/cm^3^) until the higher density mineral sank into the balloon and the mineral with lower density floated. The density of the quartz is 2.65 g/cm³, and the feldspar varies from 2.55 to 2.63 g/cm³ [21]. In the separation process, an intermediate layer was formed, which was discarded in the study.

### 2.3. Methods

Rocks samples were characterized by using chemical analysis and petrographic analysis. Whole rock chemical analysis was performed in an X-ray Fluorescence Spectrometer Rigaku X RIX 2000 (Rigaku, Austin, TX, USA) from Geoscience Institute of Federal University of Rio Grande do Sul (BR). Petrographic analysis by optical microscopy was performed together with the quantitative modal analysis in order to quantify each different quartz microstructure, which reflects the different degrees of deformation of the quartz. The thin section description was made with assistance of an optical microscope model Leica-DM4500 P LED (Leica, Frankfurt, Germany) and the optical images were obtained using the digital camera Leica Model DFC-495 (Leica, Frankfurt, Germany).

To evaluate the development of ASR within the samples, the accelerated mortar bars test, recommended by ASTM C 1260 [22], was used. After the test, the mortar bars were sawn, fixed in glass, and polished, to a thickness of approximately 30 μm, for analysis by optical microscopy, in order to observe the silica dissolution after the alkaline attack, as well as the distribution of the ASR gel.

The NBR 9848 test [23] was performed to evaluate the silica dissolution from the quartz. The assay consists of determining the coloration resulting from a silico-molybdic complex with a pH close to 1 by visible spectrophotometry. The silica concentration was determined in the water collected from the filtration of the samples subjected to the alkaline attack (1 M KOH solution for 3 days, 80 °C).

Nuclear magnetic resonance spectroscopy (NMR) measures the intensity of absorption or emission of electromagnetic radiation at certain frequencies. When the technique is used to evaluate solids, it is possible to determine the structure of the compounds. To measure an NMR spectrum, a reference material is used as the standard. In this study, the frequency range studied was ^29^Si, to verify on the atomic scale the influence of the quartz deformation structures on the susceptibility of ASR occurring. Samples were evaluated before and after the alkaline attack in a 1 M solution (KOH) for 3 days at 80 °C.

The chemical shift (δ) from the NMR to the silicate group is determined by the number of Si–O–Si and/or Si–O–Al bridges, in addition to weaker perturbations that are produced by cations coordinating the other oxygen atoms present. The tetrahedra species are identified with the nomenclature QnmAl, where n is the number of oxygens in Si–O–Si bridges (n = 0, 1, 2, 3 or 4) and m the number of oxygens in Si–O–Al bridges.

In order to verify if the separation of the quartz from the different rocks (with different intensities of deformation) was carried out appropriately, i.e., to verify if there is predominance of quartz after minerals are separated, X-ray diffraction (XRD) with Rietveld refinement was carried out. The data acquisition time was 16 s at each step of 0.02°, from 2 to 72° (2θ scale). A Siemens Bruker-AXS diffractometer (Bruker, Massachusetts, USA), model D5000 with goniometer θ-θ was used, with Cu–Kα radiation (λ = 1.5418 Ǻ) being the graphite monochromator. The operating conditions of the X-ray tube were 40 kV and 25 mA.

Scanning Electron Microscopy (SEM) was carried out in a Jeol 6610-LV (Hitachi, Tokyo, Japan) at the Isotopic Laboratory of the Geosciences Institute of Federal University of Rio Grande do Sul (BR). Backscattered Electron Imaging combined with energy dispersive spectrometry (EDS) were employed to assess the compositional change of quartz crystals after the alkaline attack. For SEM-EDS analysis, thin laminates of the mortars submitted to the ASTM C 1260 accelerated assay were used in order to evaluate the dissolution of the aggregates used in the accelerated test.

## 3. Results

### 3.1. Chemical and Petrographic Characterization of the Rocks

Rock chemical analysis (Table 1) show the high SiO_2_ content of the granite and the mylonites and that the whole composition of the rocks is not greatly affected by the deformation process, which is confirmed by the petrographic observations.

Mineralogical composition of M1, M2, and M3 was identified, characterized, and quantified qualitatively through petrographic analysis.

According to the petrographic analysis, M1 is a coarse-grained monzo to sienogranite, with a porphyritic, hypidiomorphic texture. The K-feldspars megacrystals has an average size of 3.0 cm, reaching 8.0 cm. The matrix comprises quartz (37.6%), K-feldspar (33.8%), plagioclase (25.2%), and biotite (3.5%) with sizes between 0.1 and 1.0 cm. As accessory minerals, zircon, epidote, allanite apatite, and opaques were identified.

**Figure 1:**
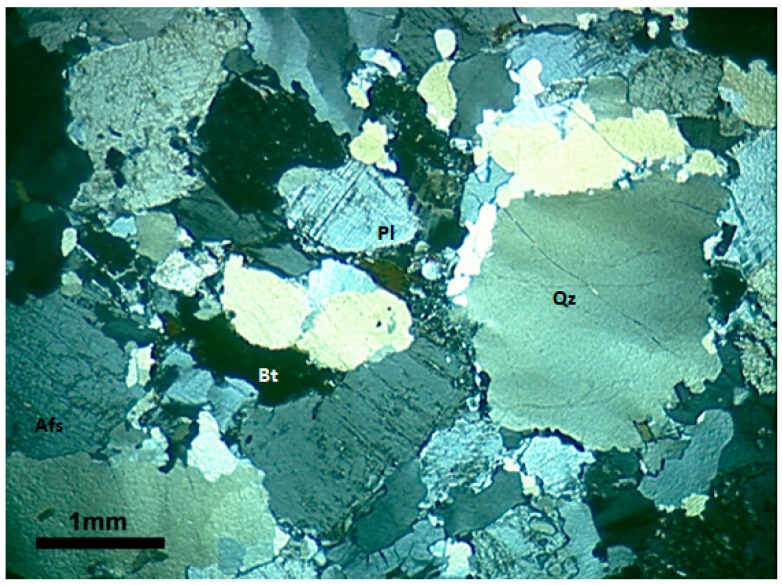
General aspect of the matrix of granite M1 subjected to low deformation rates. Microstructures observed in quartz grains are mainly undulatory extinction, deformation bands, and the formation of subgrains, especially in the large igneous quartz grain boundaries. Optical microscope under crossed polarizer. Afs = K-feldspar, Qz = quartz, Pl = plagioclase, and Bt = biotite.

The quartz of M1 is characterized by the weakest deformation among the three rocks analyzed. It appears with anhedral grains with sizes between 0.2 and 0.6 cm. These crystals invariably present deformation features such as undulatory extinction and, in a few regions, deformation bands and subgrains, as shown in Figure 1. The undulatory extinction and the deformation bands occur in the larger crystals and become diffuse in the vicinity of the grain boundaries where the deformation bands are replaced by small subgrains. Dynamic recrystallization features, characterized by the appearance of small recrystallized grains, occur in the periphery of the grains.

**Figure 2:**
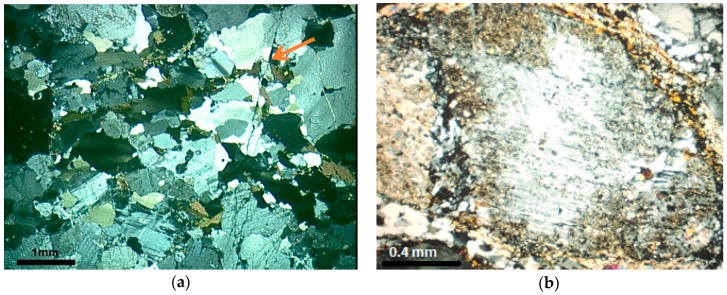
(**a**) General aspect of the protomylonite M2. Notice the finer and more homogeneous grain size of the rock and a spaced foliation marked by the slight orientation of platy biotite grains and stretching of quartz (arrow). Undulatory extinction and deformation bands are observed in coarser grains and smaller new grains; (**b**) Detail of high strain zone in protomilonite. Observe plagioclase porphyroclast with rotation fracture and core-and-mantle structure filled by fine-grained recrystallized albite. Quartz fine-grained aggregates surround feldspar. Optical microscope under crossed polarizer.

Sample M2 represents the transition from granite to mylonite, with decrease in grain size, definition of a spaced foliation marked by the slight orientation of platy grains and stretching of quartz (Figure 2a). The mineral composition consists of quartz (28.7%), K-feldspar (39.6%), plagioclase (26.3%), and biotite (5.4%). The M2 rock, according to the Wise classification [24], corresponds to a protomylonite. Quartz of this rock has intermediate deformation, among the three rocks analyzed. The mineral begins to acquire more elongated forms, which are stretched according to the foliation, with strong undulatory extinction and bands of deformation. Small bands of fine-grained recrystallized quartz are formed, which are in irregular arrays with serrated contacts.

In M2, there is a reduction in the size of the grains of quartz, which are more uniform in this rock. However, they sometimes appear as large crystals (1.0 to 2.0 mm), but with deformation features, such as undulatory extinction, deformation bands, subgrains, and recrystallized crowns of quartz around the larger grains. Quartz ribbons begin to appear, separated from the other minerals of the rock. In these places, the grains are more stretched and can present features that range from intense deformation, with strong undulatory extinction and subgrains, to mosaics of new grains, surrounding feldspar porphyroclast, without undulatory extinction and which are free of deformation (Figure 2b).

**Figure 3:**
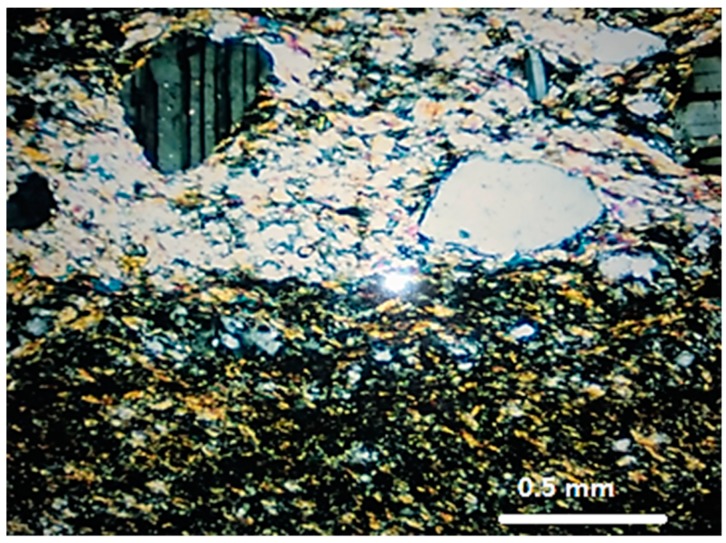
General aspect of the mylonite M3. Segregation of micaceous and quartz-feldspatic bands. Quartz in fine new grains aggregates. Optical microscope under crossed polarizer.

Sample M3 is a mylonite [24], composed by quartz (33.2%), K-feldspar (36.5%), plagioclase (24.0%), and biotite (6.3%). The quartz of M3 still preserves some large grains with undulatory extinction and subgrains, but the main feature is the presence of aggregates resulting from the stretching and reorganization of the grains in the form of ribbons, with progressive segregation of the minerals, forming alternating micaceous and quartz-feldspatic bands that define the stronger mylonitic foliation (Figure 3). The quartz grains of M3 are the most deformed among the three rocks evaluated.

The quartz-feldspatic bands are mainly characterized by aggregates of new small grains (<0.3 mm). These new grains can have contacts that range from those which are serrated and interpenetrating, with strong preferential orientation and, moreover, deformed, with undulatory extinction, to straighter, polygonal contacts without preferential orientation, which are free of deformation.

As shown by Tiecher et al. [6], five stages of deformation of the quartz grains can be individualized, which were later quantified in each of the rocks, as shown in Table 2. The stages themselves are defined as follows: 

- Stage 0: deformation-free grain

Quartz free of defects, without deformation. The quartz grain is characterized by the absence of undulatory extinction, i.e., it extinguishes perfectly the light when the stage of the optical microscope is rotated, which shows that its crystalline reticulum is free of defects (displacements).

- Stage 1: grain with undulatory extinction

The optical characteristic of the mineral in this stage indicates that there is deformation of the crystalline reticulum. On observing quartz through petrographic microscopy, the light does not extinguish in a homogeneous way but has lighter and darker areas.

- Stage 2: grain with undulatory extinction which forms deformation bands

Deformation increases and the undulatory extinction becomes more pronounced, creating well-defined zones within the crystal (the beginning of the formation of the displacement walls). These zones/bands are called deformation bands and represent regions where the grain is more fragile.

- Stage 3: grain with undulatory extinction which starts to form subgrains

When deformation increases, the deformation bands are amplified, i.e., the defects in the crystalline reticulum of the mineral grow, thereby creating regions where the chemical bonds tend to break more easily. Since subgrains have well-defined walls, this leads to their developing more easily within the grain of origin.

- Stage 4: recrystallized grain

In the last stage of the deformation process, subgrains are completely separate and form new grains without defects in a process called recrystallization. Recrystallized grains have the same characteristics as those of Stage 1, i.e., without deformation of the crystalline reticulum, but they are of smaller size.

### 3.2. Relationship between the Deformation of the Quartz and Susceptibility to the Alkaline Attack

The influence of textural characteristics of quartz to AAR was evaluated isolating this mineral, i.e., quartz grains were extracted from the matrix, as described in Section 2.2. Analysis by XRD, together with Rietveld’s refinement, showed that the separation procedure of the minerals obtained satisfactory results, according to the GOF (goodness of fit) values obtained (Table 3). However, as can be seen in Table 3, other minerals were also identified in very small proportions (albite, microcline, and muscovite). This was expected, since there are particles where different minerals get together during grinding. The sample with higher “contamination” of other minerals was QM3 (quartz extracted from sample M3), in with the quartz grains is smaller.

**Figure 4:**
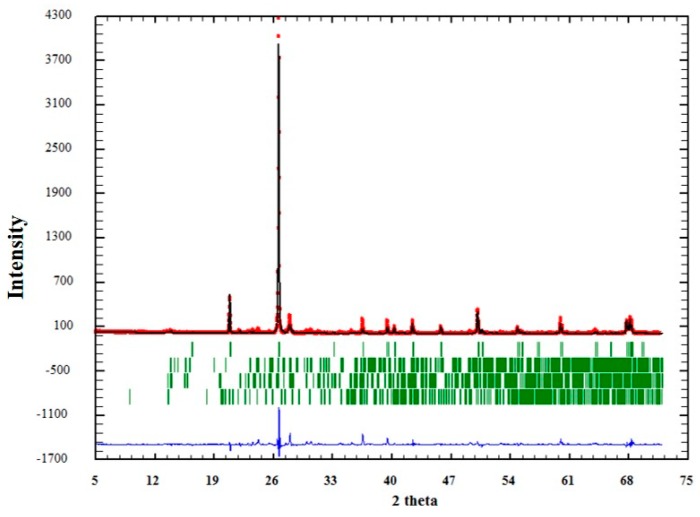
Adjustment through the Rietveld refinement of sample QM1.

**Figure 5:**
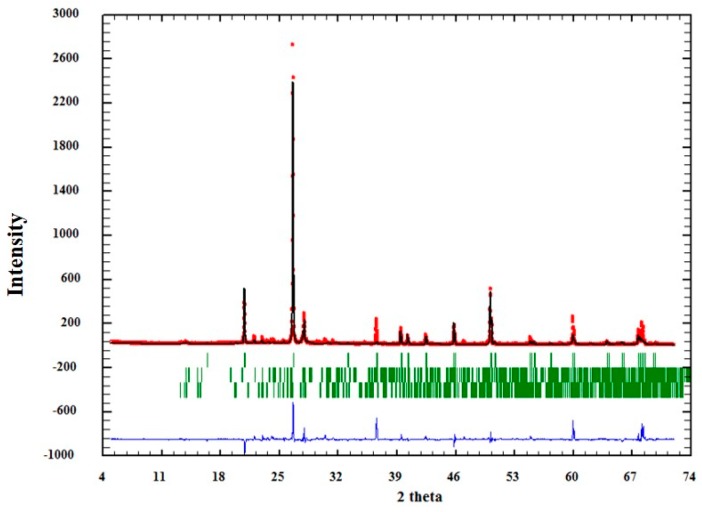
Adjustment through the Rietveld refinement of sample QM2.

**Figure 6:**
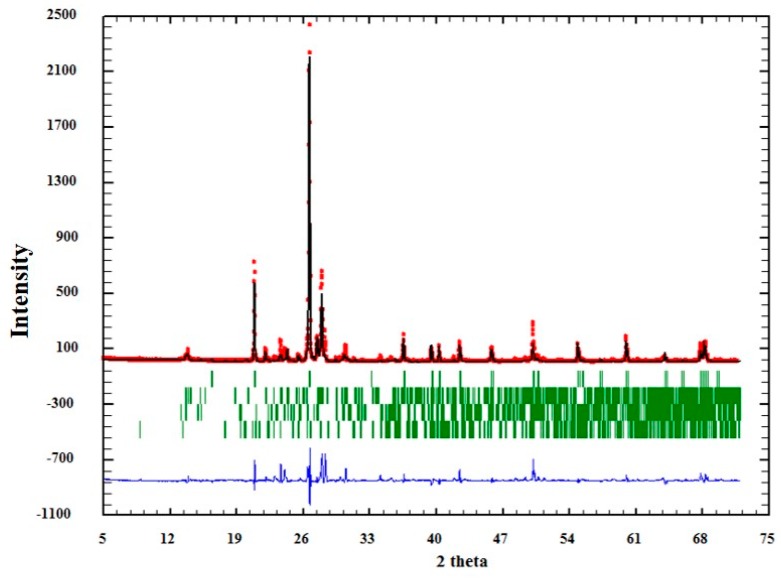
Adjustment through the Rietveld refinement of sample QM3.

The quality of the analysis by Rietveld can also be observed graphically in Figure 4, Figure 5 and Figure 6. In them, the red dots represent the data obtained experimentally, and the black line denotes the data calculated by the refinement. The error of the analysis is expressed by the blue line.

**Figure 7:**
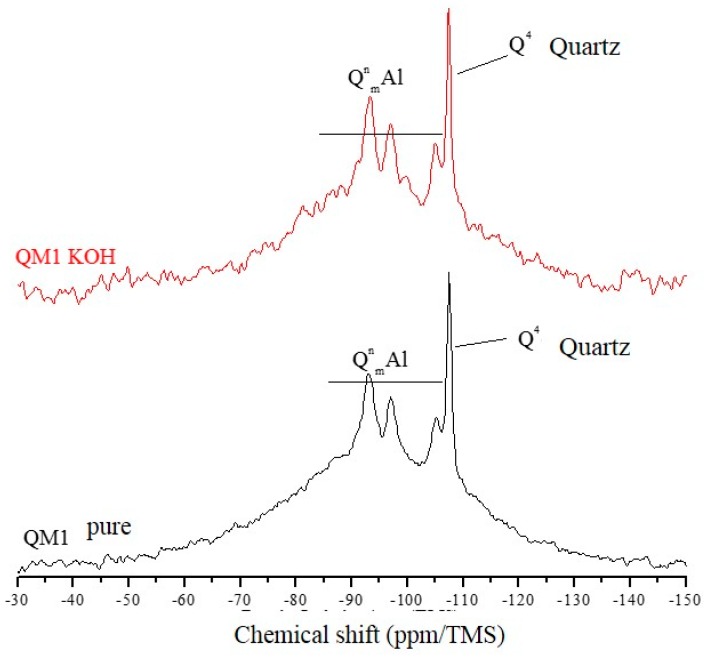
Spectra obtained by NMR using direct polarization of ^29^Si, of the QM1 sample.

**Figure 8:**
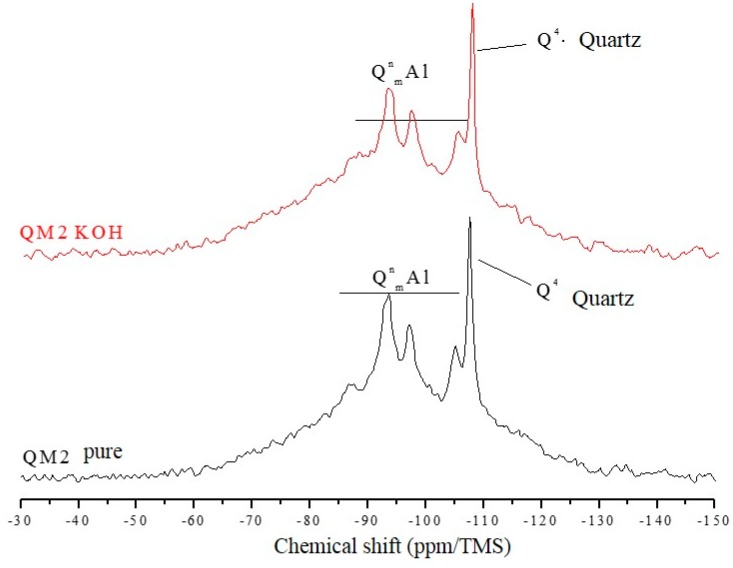
Spectra obtained by NMR using direct polarization of ^29^Si, of the QM2 sample.

**Figure 9:**
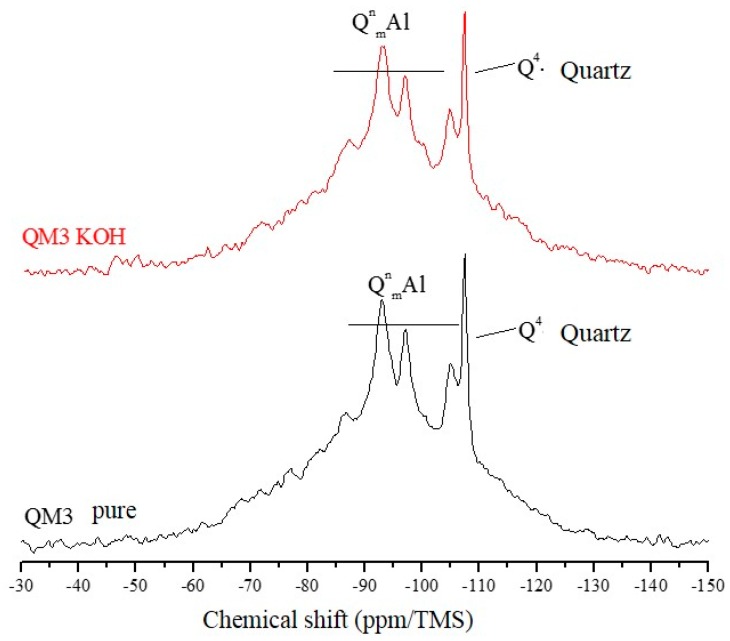
Spectra obtained by NMR, using direct polarization of the ^29^Si, of sample QM3.

The ^29^Si NMR spectra of Figure 7, Figure 8 and Figure 9 are relative to the QM1, QM2, and QM3 samples, respectively. These spectra show what has already been verified using Rietveld’s refinement, i.e., all the quartz extracted contain small contamination of the microcline, albite, and micas (muscovite). Through NMR, a peak at −107.4 ppm was identified, which corresponds to a crystalline Q4 site (crystalline quartz). It was also possible to recognize, in all spectra, narrower sites at −93.4, −97.3, and −100.2 ppm, associated, in the literature, with silicates containing potassium and aluminum [25], such as K-feldspars (KAlSi_3_0_8_). The peak at −105.2 ppm corresponds to phases containing sodium and aluminum, i.e., plagioclase (NaAlSi_3_O_8_) and the narrow peak at −87.4 ppm is indicative of the presence of muscovite ((Ca, Na) (Mg, Fe, Al, Ti) (Si, Al)_2_O_6_). All peaks referring to the Si–O–Al sites are grouped in the spectra and identified by the QnmAl nomenclature.

All NMR spectra of the pure samples and KOH-attacked samples, for which there is a wide base, corresponding to quartz in an amorphous network [26], which in this analysis means the presence of quartz of nanometric dimensions, and small percentages of muscovite. NMR showed that grains classified as slightly deformed in optical microscopy, which were predominant in the QM1, on the atomic scale, are comparable to the most deformed grains, which deform to the extent that they form stretched grain clusters (deformation bands), predominant in the QM2 sample. It is understood that the so-called amorphous sites in NMR are those which represent nanometric grains of silicates present in the sample. The crystalline sites, on the other hand, do not correspond to perfectly crystallized grains, but rather to larger and more stable grains.

In the amorphous portion of the spectra of all samples, the Qn sites have different Si–O–Si connectivities, corresponding to the species Q0, Q1, Q2, Q3, and Q4, similar to what the study by Leemann [27] concluded. Florindo [28], in a study of samples of pure granite and attacked with KOH (there was no separation of the quartz), verified that the structure of the rock essentially consists of Q4 and Q3 sites, with reference to quartz and muscovite. The KOH attack promoted the increase of the most connected sites (Q1 and Q4).

NMR analyzes performed by Tambelli et al. [29] show that the atomic structure of the ASR gel has silicate connectivity similar to Q3. On the other hand, in the study of Cong et al. [30] it is verified that for the formation of the ASR gel, in the presence of calcium, Q4 sites predominate. The Q1 sites are less abundant and partly transient. The gel produced in the absence of calcium has large peaks, corresponding to the sites Q3, Q2, and Q1.

The Qn site presents a distribution of isotropic chemical shifts caused by the 29Si structural disorder, which gives rise to Gaussian-type line shapes in the NMR spectra. The groups Q0, Q1, Q2, Q3, and Q4 may be present in amorphous or crystalline areas (in the form of more pronounced, narrow lines in the spectra). The Q4 groups, which form the structure of the silica (quartz), have a chemical shift with values around −110 ppm. Studies evaluating the products formed by the ASR show that it has a layered structure [31].

Table 4 shows that the percentage (in area) of amorphous sites (nanoscopic grains) is well above that of crystalline silicate sites in all samples. The crystalline quartz sites represent only a small part of the area of the crystalline silicates present. This shows, once again, that even grains that have little deformation in the optical microscopy (predominant in sample QM1, for example), have displacement walls which form nanometric subgrains, i.e., they have fragile zones that can react with the alkaline hydroxides over time. Table 4 also shows that there was a reduction in the percentage of crystalline silicate sites after the attack for all samples, as well as an increase in the percentage of amorphous sites. For QM2, the reduction of the crystalline quartz sites after the KOH attack was more intense, of the order of 10%, followed by QM1, which was reduced by 6.5% and QM3, which was reduced by 1.9%.

It is important to point out that these results could not be taken as true if they were obtained alone, but only after NMR analysis, since the differences observed between the samples before and after the KOH attack were small. However, there is support for the other results obtained in the study, such as the analysis by using the visible spectrophotometer method [23], which evaluated the relationship between silica dissolution by the alkaline attack and the different stages of deformation of the quartz. The results are presented in Table 5.

**Figure 10:**
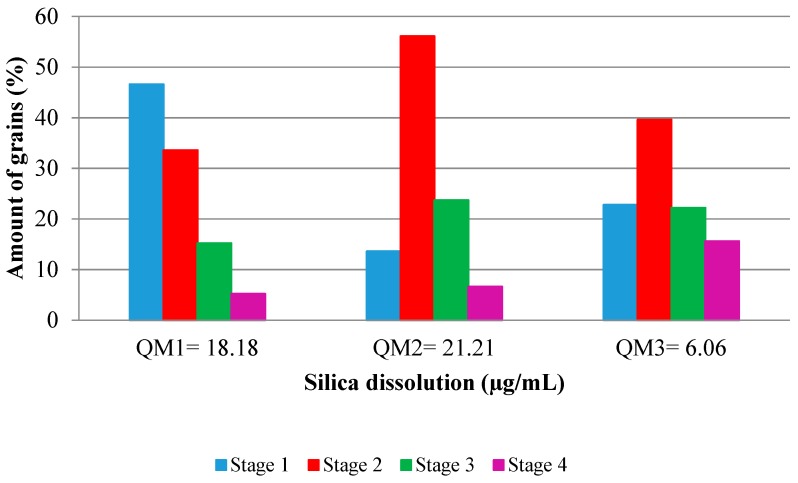
Relationship between silica dissolution and the deformation of the quartz.

It is considered that the higher the silica dissolution, the higher the incidence of ASR. According to Figure 10, the grains with Stage 2 (quartz deformed with stretched feature in bands) dissolve more intensely, which agrees with the Stastná et al. [32]. These are the predominant grains in M2. The grains classified as slightly deformed (Stage 1), which exhibit slight undulatory extinction in optical microscopy, were very susceptible to dissolution. The QM1 sample presented higher dissolution than the more deformed sample, QM3. QM3 showed little susceptibility to the silica dissolution, when compared to the others, showing that the deformation in Stages 3 (subgrain) and 4 (recrystallized) is similar to the development of ASR.

### 3.3. Assessment of the Development of the ASR

**Figure 11:**
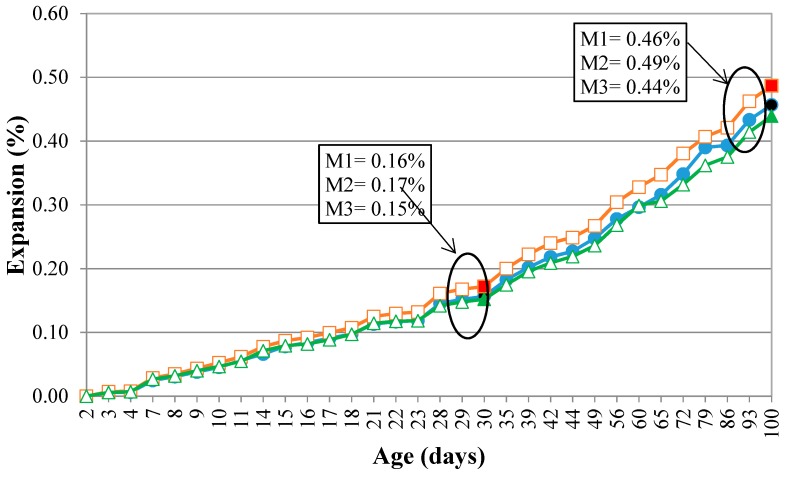
Expansion of the M1, M2, and M3 in the mortar bar accelerated test.

Figure 11 shows the expansions from the accelerated mortar bar test. It was verified that, at around 30 days, the mortars expand by more than 0.10% and less than 0.20%. This behavior is quite common when the silica involved in the reaction comes from the deformed quartz because this mineral takes longer to react with the alkali hydroxides [33,34]. It is also observed that the curves of the expansions over time denote a behavior with continuous growth of the expansions to the three rocks, with no tendency to stabilize until the samples have been exposed to the alkaline solution for 100 days. This suggests that caution needs to be exercised when using these aggregates, since the behavior presented in the 30 days of the test may have been masked by the slow development of the reaction by the quartz grains.

The expansions measured for M3 were lower than the other samples. M3, as shown previously, is the rock with the greatest deformation, which contains predominantly recrystallized quartz grains (Stage 4), these grains do not have deformation of the crystalline reticulum, that is, they take longer to react (Figure 11). Figure 11 shows that the differences between the expansions increase with the passage of time, probably because, at first, the reaction with the alkali hydroxides occurs between quartz with intense undulating extinction, forming bands (Stage 2). Afterward, the grains of Stages 1 and 3 begin to react, which have very similar crystallographic characteristics, despite different sizes.

In order to evaluate if the differences between the expansions of the M1, M2, and M3 rocks can be considered significant, it was take the variance analysis (ANOVA) of the influence of the type of the rock on the expansions test was performed (Table 6).

According to Table 6, expansions from the mortar bar test can be considered statistically different for M1, M2, and M3. Based on Test F and the *p*-value, it was verified that the type of rock influences significantly the expansions, as do the age and the interaction between the type of rock and age. Thus, M2 expanded/reacted more intensely than M1, which expanded more than M3, in the same way it was observed in the NMR and by the visible spectrophotometer (silica dissolution analyses). Slightly deformed quartz, predominant in the M1, effectively contributes to the occurrence of ASR, together with the grains of Stage 2, which are also expressive in this sample.

In the sample M3, the percentage of grains with Stage 2 are 15% higher than M1, but expansions measured for M3 were lower. The recrystallized grains (Stages 3 and 4, predominant in M3) are less susceptible to the alkaline attack. Grains of the Stages 3 and 4 result from more intense deformations undergone by the rock. However, their expansions were less than the others’.

**Figure 12:**
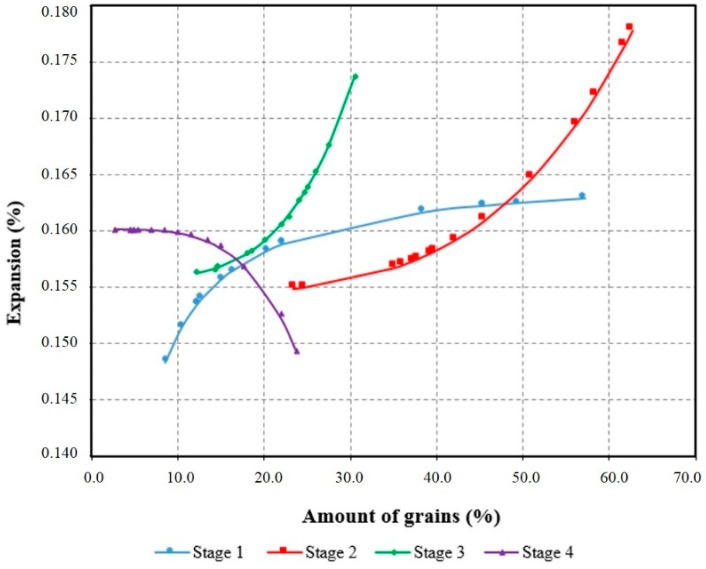
Representative curves of the model obtained model to evaluate the influence of the stages of deformation of the quartz in the expansions of the ASR.

Based on the quartz with different stages of deformation which were quantified in this study and the results of the accelerated mortar bar test at 30 days, it was possible to obtain a model of the behavior (Equation (1)), which represents 70% of the experimental data (R^2^). From this model, the expansions were recalculated and results were obtained by varying each stage of deformation alone and keeping the other stages constant. Thus, it was possible to trace the behavior curves for the degrees of deformation, as shown in Figure 12.

(1) $$\mathrm{Expansion}=\;\left(0.156516\;-\;\frac{0.147477}{\mathrm{Stage}\;1}\right)\;+\;\left(\left({0.151112\;E}^{-9}\right){\;\times \;(\mathrm{Stage}\;2)}^{4}\right)\;+\;\left(\left({2.06331\;E}^{-8}\right)\;\times \;\;{\left(\mathrm{Stage}\;3\right)}^{4}\right)\;-\;\left(\left({3.40005\;E}^{-8}\right)\;\times \;{\left(\mathrm{Stage}\;4\right)}^{4}\right)\;$$

Correlation coefficient (R²) = 70%.

The curves obtained by Equation (1) are valid only to interpret the behavior of the expansions arising from M1, M2, and M3. The results are similar to those of the assays. Thus, Figure 12 shows that the smaller expansions are due to the presence of grains with little deformation (Stage 1) and of recrystallized grains (Stage 4). It shows too the importance of Stages 2 and 3 for the growth of the expansions. The curve obtained for Stage 4 shows that the recrystallized grains do not represent a risk of ASR occurrence. However, the variation in the amount of grains for this stage of deformation (from 2% to 23%) was very small.

**Figure 13:**
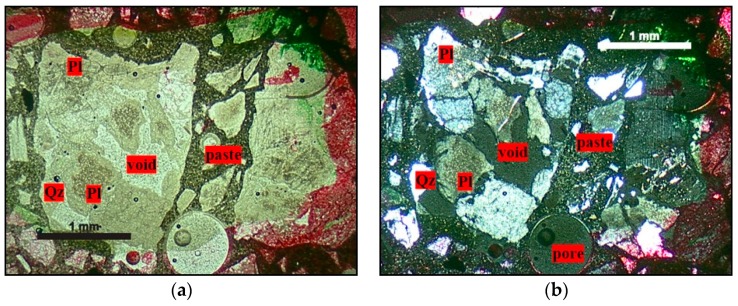

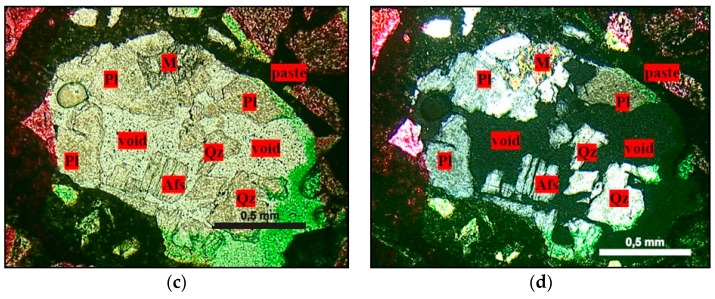
Micrographs obtained by optical microscopy showing quartz dissolution in mortars submitted to the accelerated mortar bar test, where Qz = quartz, Pl = plagioclase, Afs = K-feldspar, and M = mica. (**a**,**b**) Natural light and crossed polarizer, 2.5× magnification; (**c**,**d**) crossed polarizer, 5× magnification.

A petrographic analysis was performed on mortars submitted to the accelerated mortar bar test, where it was verified that the alkaline solution effectively dissolves the quartz first. Figure 13 shows that the feldspars and micas are not dissolved by the alkaline solution.

**Figure 14:**
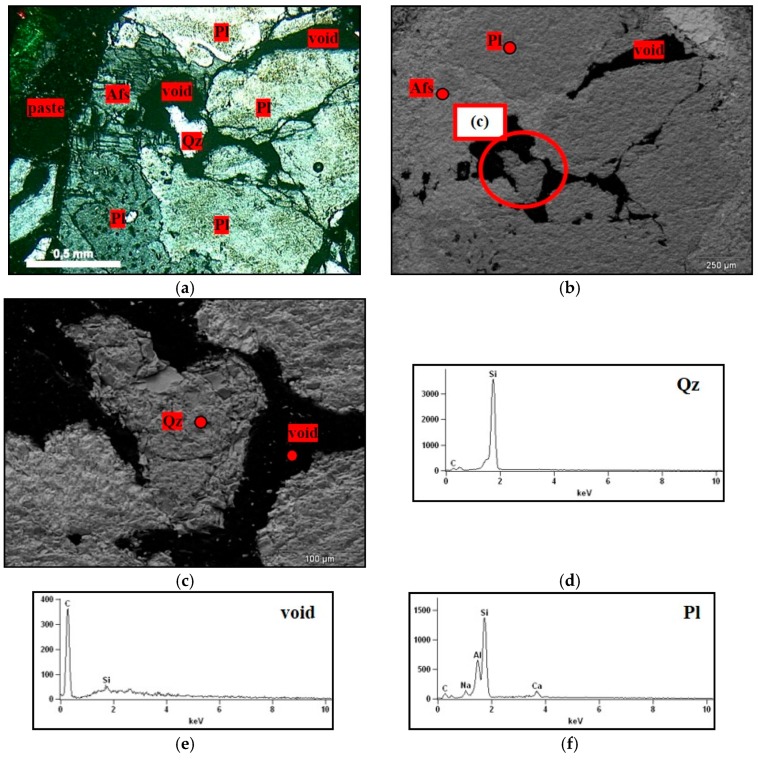

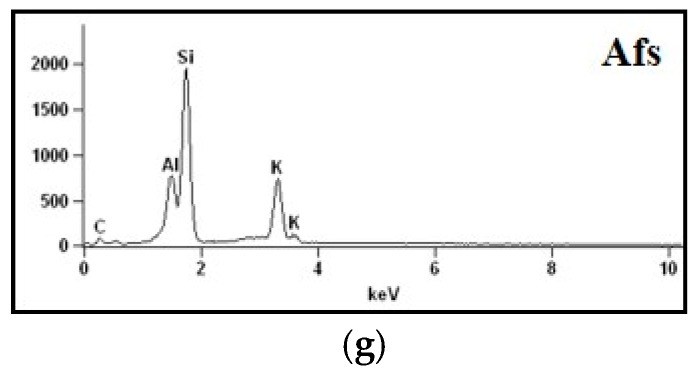
Fragment of the rock in the mortar submitted to the mortar bar test, where: Afs = K-feldspar, Pl = plagioclase, and Qz = quartz. (**a**) Micrograph obtained by optical microscopy under crossed polarizer of the rock showing voids because of the dissolved quartz, 5× magnification; (**b**,**c**) micrographs obtained in SEM detailing quartz not yet dissolved, 75× and 300× magnification; (**d**–**g**) spectra obtained by EDX from quartz, void, plagioclase, and K-feldspar.

SEM with EDX enabled it to be verified that there was a predominance of the plagioclases and K-feldspars in the fragments exposed to the alkaline solution (Figure 14a,b,f,g). In Figure 14c,e, there are also voids within the grains of the M1, M2, and M3 rocks, which correspond to the quartz dissolved due to ASR.

**Figure 15:**
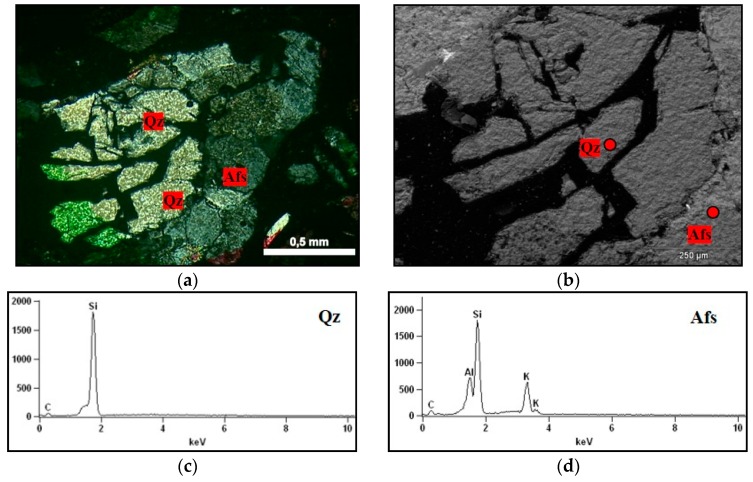
Fragment of the rock in the mortar submitted to the mortar bar test, where: Afs = K-feldspar, Qz = quartz. (**a**) micrographs obtained under optical microscopy under crossed polarizer of the rock showing voids inside the grain of quartz, 5× magnification; (**b**) micrograph obtained in SEM detailing the presence of partially dissolved quartz grains, 130× magnification; (**c**,**d**) spectra obtained by EDS of the quartz grain, and K-feldspar.

On observing the rock fragments from the accelerated mortar bar test, partially dissolved quartz was detected, which presented voids within the grain itself. This situation is illustrated by Figure 15, which shows that the alkaline solution penetrates the more fragile regions of the quartz (probably in the displacement walls of the grain). Similar characteristics were observed by Mo et al. [35]. However, over time, all quartz tends to dissolve and to contribute to ASR.

**Figure 16:**
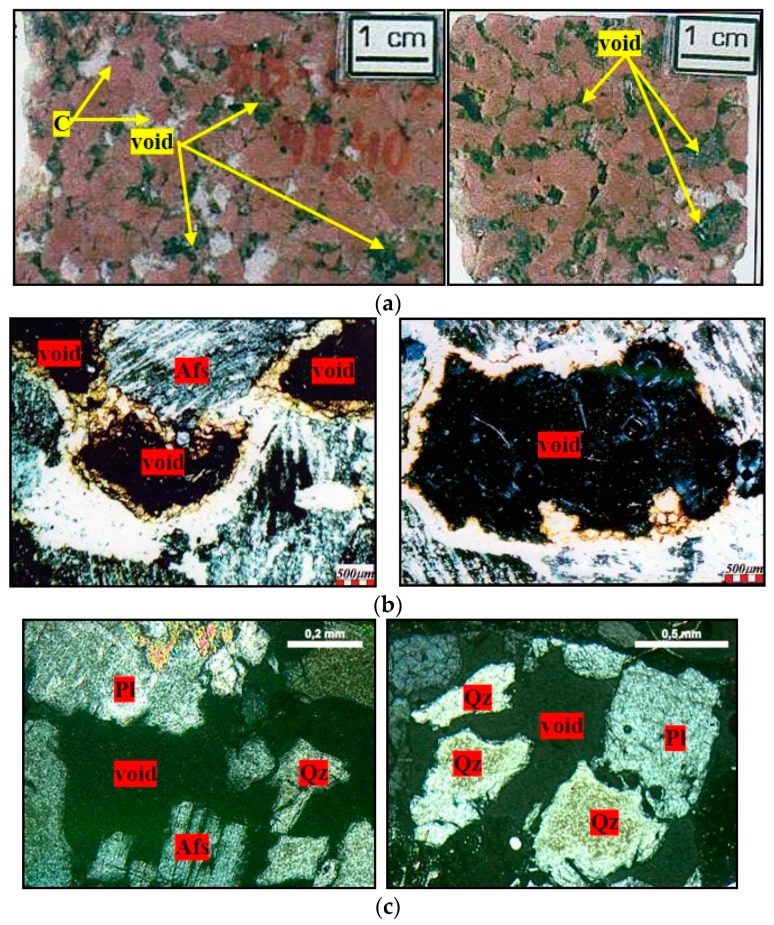
Quartz dissolution by alkaline fluids, where Afs = K-feldspar, Pl = plagioclase, C = carbonate, and Qz = quartz. (**a**) Photographs of the epissienite rock [36]; (**b**) micrographs obtained by optical microscopy under crossed-polarized of the epissienite [36]; (**c**) micrographs obtained by optical microscopy under crossed-polarized of rock fragments after the accelerated mortar bar test, 5× magnification and 10× magnification.

The dissolution of the quartz in the mortars resembles what occurs in nature, when alkaline fluids come into contact with acidic rocks. Mexias [36] reveals the feature of epissienites, which are rocks originating from the hydrothermal alteration of granites, with voids, hitherto occupied by quartz, as shown in Figure 16a. Figure 16b shows the characteristics of the rock under optical microscopy, which can be compared with Figure 16c, which shows rock fragments from the accelerated mortar bar test of the present study.

## 4. Discussion

In the literature, numerous studies observe the relationship between rock characteristics and their susceptibility to silica dissolution [7,8,37,38]. Locati et al. [37] found that quartz from milonitized rocks were 97% more reactive than non-milonitized ones due to the presence of grains that were intensely deformed to form subgrains.

Ponce and Batic [7] verified that the texture of the minerals changes the manifestations of the ASR. Leemann and Holzer [8] showed that quartz is the most susceptible mineral to alkaline attack and, observing polished sections, verified different morphologies for ASR gel, according to the texture of this mineral. According to Marinoni et al. [38], when the alkaline attack occurs, first there is the dissolution of the quartz belonging to the aggregates followed new crystals precipite.

The main contribution of the present study is the evaluation of the silica dissolution, promoted by the alkaline attack, made using quartz extracted from the rocks. Samples evaluated have quartz with different stages of deformation; thus, it was possible to verify the importance, for the dissolution of silica, of intensely deformed grains, forming bands and subgrains (Stage 2). It has also been observed that recrystallized grains contribute less to the dissolution of silica.

**Figure 17:**
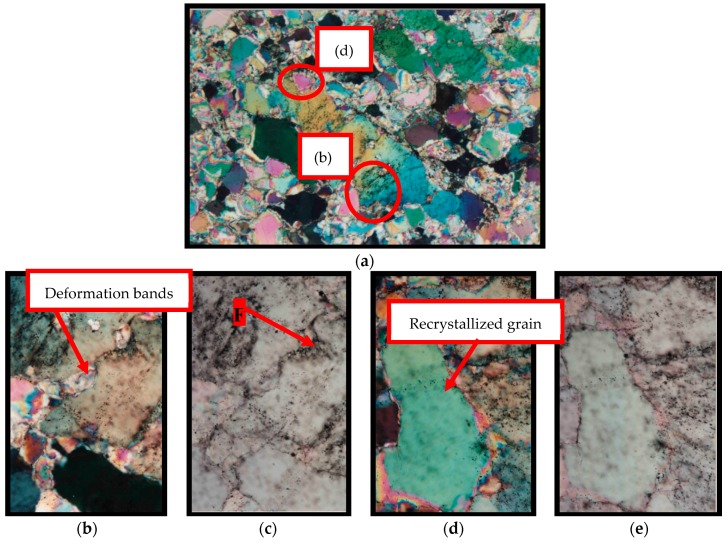
Micrographs obtained by optical microscopy evidencing the presence of fluid inclusions in the deformed quartz, where F = fluid. (**a**) Micrograph of the region containing deformed and recrystallized quartz, crossed-polarized; (**b**,**c**) deformed quartz with inclusions in deformation bands, crossed-polarized and natural light; (**d**,**e**) recrystallized quartz without fluid inclusions, crossed-polarized and natural light [18].

A good example of how alkaline hydroxides penetrate into the quartz grains, promoting ASR, is illustrated below by the micrographs published by Mesquita [18]. The author studied the metamorphic-hydrothermal alteration of quartz in nature and verified that the presence of displacement walls in the grains allows the entry of quartz-altering fluids (Figure 17b,c). However, in recrystallized grains, no fluid inclusions are observed (Figure 17d,e); that is, in these grains there are no paths for the penetration of fluids.

## 5. Conclusions

In order to better explain the relation between the quartz characteristics for the development of ARS, four stages of deformation were established by using petrographic analysis. The analysis was performed to evaluate the silica dissolution of the different textures of quartz performed on the grains extracted from the rocks. This showed that the presence of displacement walls in the quartz (Stage 2) facilitates the reaction with the alkali hydroxides. The displacement walls are regions in the grains where the chemical bonds between Si and O are brittle and can be viewed under optical microscopy. The same behavior was observed when the evaluation was made using the accelerated mortar bar test in which the rock which was used did not have the extracted quartz.

Using NMR analysis by direct polarization of ^29^Si, it was possible to verify that the poorly deformed quartz (Stage 1) shows the same characteristics as the most deformed grains (Stages 2 and 3) do on a molecular scale. In addition, the analyses indicated that, after the alkaline attack, there is a greater reduction in the number of crystalline sites in sample M2, in which there are largely grains of Stage 2, thus suggesting that Stage 2 can react faster than Stages 1 and 3, respectively, thereby contributing more to the occurrence of the ASR.

Further investigations will be performed using XANES (X-ray Absorption Near Edge Structure) and EXAFS (Extended-Ray Absorption Fine Structure), through the Synchrotron Energy, both in grains extracted from rocks and in grains of pure synthetic quartz. The synthetic quartz will be used as a reference parameter in order to evaluate whether the results obtained in the extracted samples are influenced by the presence of micro impurities present.

## Figures and Tables

**Table 1:** Chemical composition of rocks.

**Compound**	**M1**	**M2**	**M3**
	**Major Elements (% Mass)**		
SiO_2_	78.18	77.46	77.33
Al_2_O_3_	11.82	12.16	12.02
TiO_2_	0.10	0.13	0.14
Fe_2_O_3_ (total)	1.74	1.58	1.66
MnO	0.03	0.04	0.04
MgO	0.05	0.13	0.16
CaO	0.78	0.89	1.05
Na_2_O	1.98	2.14	2.14
K_2_O	5.12	5.06	4.63
P_2_O_5_	0.02	0.02	0.01
LOI	0.24	0.27	0.24
TOTAL	100.07	99.88	99.42
	**Trace Elements (ppm)**		
Y	6	6	5
Pb	47	47	45
Ni	-	-	-
Co	-	-	-
Cu	8	9	9
Ga	11	13	12
Sr	118	109	158
Zr	78	78	81
Zn	18	24	27
Nb	2	5	3
Rb	198	205	186
As	4	4	3
Cr	88	28	60
Ba	491	483	618

**Table 2:** Percentage amount of quartz in different stages of deformation present in the M1, M2, and M3 samples.

Stage of Deformation	Description of the Characteristic	Petrographic Aspect	Modal (%)		
			M1	M2	M3
0	Quartz without deformation	---	-	-	-
1	Quartz with slight undulatory extinction	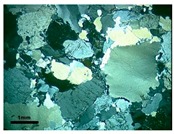	46.6	13.6	22.8
2	Quartz with strong undulatory extinction, with formation of bands of deformation in the grain	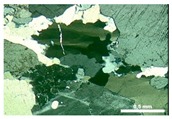	33.6	56.1	39.6
3	Quartz with strong undulatory extinction, with formation of subgrains	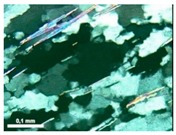	15.2	23.7	22.2
4	Quartz recrystallized from subgrain	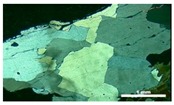	5.2	6.6	15.6

**Table 3:** Quantification of the phases present in the quartz and feldspar samples, using Rietveld refinement of XRD spectra.

Quartz Samples	Phases Identified	GOF
QM1	Quartz–95.10% Albite–2.33% Microclíne–2.43% Muscovite–0.15%	1.87
QM2	Quartz–98.51% Albite–1.21% Microcline–0.28%	1.80
QM3	Quartz–69.63% Albite–24.26% Microcline–2.01% Muscovite–4.11%	1.98

GOF (Goodness of fit) = refinement quality index-should be less than 5.00.

**Table 4:** Quantification of crystalline and amorphous silicate sites obtained by NMR.

Sample	Crystalline Silicates (% in Area)	Amorphous Silicates (% in Area)	Crystalline Quartz (% in Area)
Pure QM1	28.5	65.1	6.4
QM1 KOH	28.5	65.5	6.0
Pure QM2	30.0	63.1	6.9
QM2 KOH	29.8	64.0	6.2
Pure QM3	31.7	63.1	5.3
QM3 KOH	28.6	66.1	5.2

**Table 5:** Dissolution of silica by the NBR 9848 method.

Sample	Dissolved SiO_2_ (µg/mL)
QM1	18.2
QM2	21.2
QM3	6.1

**Table 6:** Analysis of the variance of the effects to the expansions of the ASTM C 1260 test.

Effect	Degrees of Freedom	Squared Mean	Degrees of Freedom of the Error	Squared Mean of the Error	Test F	*p*-Value
Type of rock	2	0.010758	192	0.000052	207.357	0.0000
Age	31	0.164571	192	0.000052	3174.891	0.0000
Type of rock and age	62	0.000208	192	0.000052	4.016	0.0000
